# Hydrodynamic Study on the “Stop-and-Acceleration” Pattern of Refilling Flow at Perforation Plates by Using a Xylem-Inspired Channel

**DOI:** 10.3389/fpls.2018.01931

**Published:** 2019-01-08

**Authors:** Sang Joon Lee, JooYoung Park, Jeongeun Ryu

**Affiliations:** Department of Mechanical Engineering, Pohang University of Science and Technology, Pohang, South Korea

**Keywords:** plant hydrodynamics, water transport in plants, embolism repair, water refilling, perforation plate

## Abstract

Porous structures, such as perforation plates and pit membranes, have attracted considerable attention due to their hydraulic regulation of water flow through vascular plant networks. However, limited information is available regarding the hydraulic functions of such structures during water-refilling and embolism repair because of difficulties in simultaneous *in vivo* measurements of refilling flow and pressure variations in xylem vessels. In this study, we developed a xylem-inspired microchannel with a porous mesh for systematic investigation on the hydraulic contribution of perforation plates on water-refilling. In particular, the “stop-and-acceleration” phenomenon of the water meniscus at the porous mesh structure was carefully examined in macroscopic and microscopic views. This distinctive phenomenon usually occurs in the xylem vessels of vascular plants during embolism repair. Based on the experimental results, we established a theoretical model of the flow characteristics and pressure variations around the porous structure inside the microchannel. Perforation plates could be speculated to be a pressure-modulated flow controller that facilitates embolism recovery. Furthermore, the proposed xylem-inspired channel can be used to investigate the hydraulic functions of porous structures for water management in plants.

## Introduction

**Figure 1:**
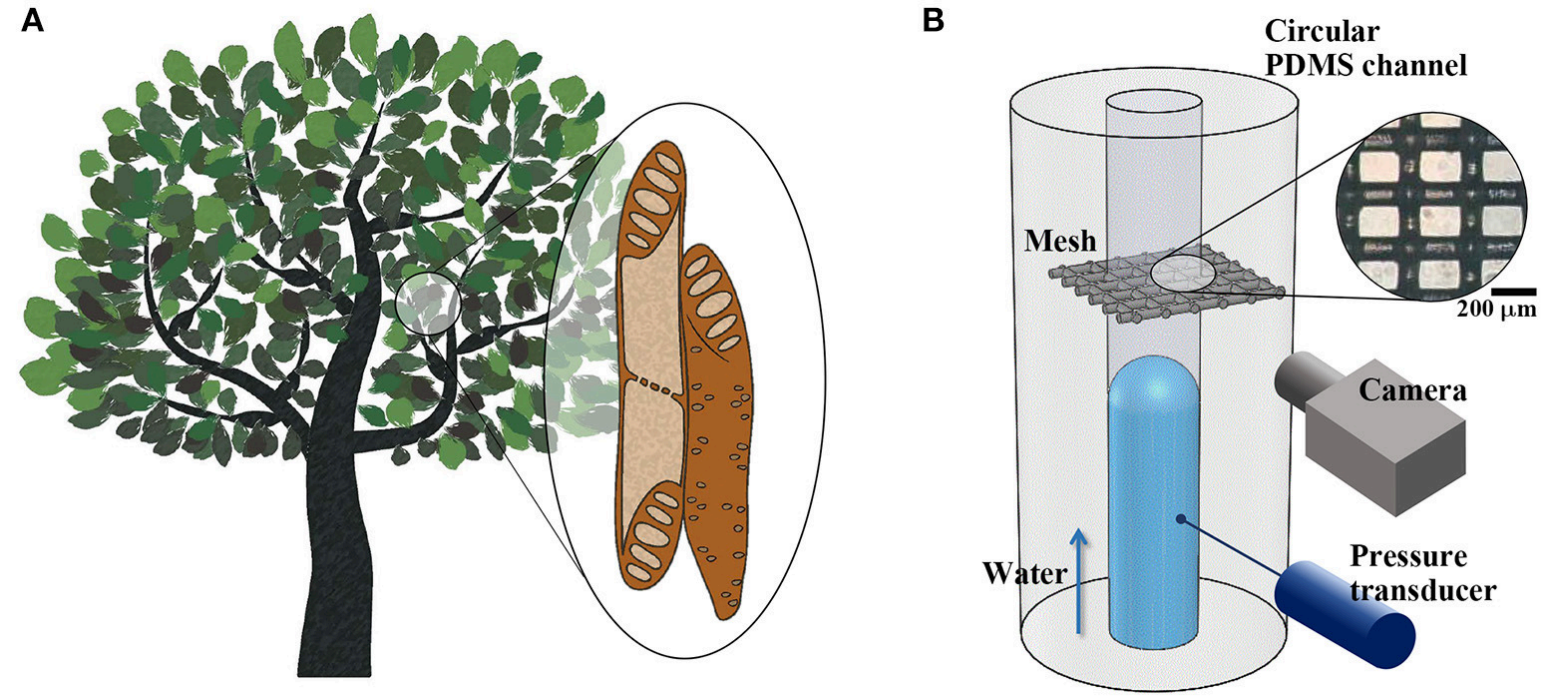
Structural features of xylem vessels of vascular plants and experimental set-up. **(A)** Schematic of xylem vessel elements. Vessel elements are interconnected through perforation plates and pit membranes located at vessel ends and side walls, constructing vascular networks. **(B)** Xylem-inspired channel and experimental setup. A piece of porous mesh was inserted between two excised cross sections of a circular PDMS channel. Side-view images of the water meniscus were obtained, and the pressure variation was measured during water-filling in the channel. Inset: Microscopic image of the mesh. Scale bar = 200 μm.

Vascular plants transport water through xylem networks, which consist of a bundle of microchannels. These channels are composed of cylindrical cells known as xylem vessel elements and contain peculiar porous structures, such as perforation plates and pit membranes (Figure 1A). Each vessel element is radially interconnected with adjacent vessel elements through pit membranes on their sidewalls. The elements are serially connected by perforation plates at the end walls. The morphological structures of the perforation plates vary depending on plant species (Meylan and Butterfield, 1975; Gevú et al., 2017). Simple perforation plates possess a single orifice-like hole at the end of each vessel element. Scalariform perforation plates contain multiple parallel slits, and reticulate perforation plates have irregular pores.

Perforation plates have been known to induce a considerable pressure drop during water transport through xylem vessels (Ellerby and Ennos, 1998). Previous studies on perforation plates have focused on the effect of the structural shapes of the plates on flow resistance in water-filled xylem vessels. A strong correlation between of the end wall resistance, vein density (Feild and Brodribb, 2013), and conduit length (Sperry et al., 2005) has been reported in terms of hydraulic efficiency in a broad range of plant species. A meta-analysis of the morphological features of perforation plates has been reported the transition from scalariform type to simple type in the evolution of woods in response to climate changes (Jansen et al., 2004; Lens et al., 2016). The hydraulic resistances of simple and scalariform perforation plates have been directly measured and compared to understand water transport in plants (Christman and Sperry, 2010). Variations in velocity field and pressure across perforation plates have been numerically estimated through simulation using model geometries with varying pore sizes, thicknesses, and perforation plate angles (Schulte and Castle, 1993a,b; Schulte, 1999). Some studies have been conducted to measure and estimate flow resistance of xylem vessel walls through large-scale physical modeling (Schulte et al., 1989; Ellerby and Ennos, 1998).

In contrast to their adverse effects on water transport, porous structures in xylem vessels can function as safety valves during cavitation (Jansen et al., 2004; Hwang et al., 2014). In general, xylem vessels are vulnerable to embolism because of the metastable state of sap as a result of negative hydrostatic pressure produced by leaf transpiration, freezing–thawing cycles, and drought stress (Brodersen and McElrone, 2013; Jensen et al., 2016). In embolized xylem vessels, perforation plates are speculated to restrain air spread toward adjacent vessel elements by trapping air bubbles (Sperry, 2013; Wheeler et al., 2013; Venturas et al., 2015) and prevent the merging of gas bubbles into a larger bubble (Sperry, 1986, 2003; Tyree and Zimmermann, 2002; Brodersen et al., 2018). In addition, perforation plates may contribute to embolism repair by dissolving gas bubbles in water through pressurizing water in refilled vessels (Lee and Kim, 2008). Recent visualization studies on embolism recovery by X-ray microimaging technique have reported the hydraulic role of perforation plates in bypassing embolized vessels through radial refilling (Lee and Kim, 2008; Lee et al., 2013; Hwang et al., 2016). However, the flow stoppage and resultant pressure variations at the perforation plates remain unclear. Thus, systematic studies on filling flow through an empty channel embedding porous structures are required to understand the hydrodynamic functions of perforation plates in embolism repair.

In this study, we demonstrated the stop-and-acceleration phenomenon of the water meniscus at a porous mesh structure installed in a microchannel inspired by the xylem vessels of vascular plants. This phenomenon occurs distinctively at perforation plates of xylem vessels during embolism repair (Jeje and Zimmermann, 1979; Lee and Kim, 2008; Lee et al., 2013; Hwang et al., 2014, 2016). Flow kinetics were analyzed by evaluating the temporal movement of the water meniscus of refilling flow based on the flow rate and pressure variation in front of the porous structure under the same flow conditions. When the water meniscus stopped at the porous structure, its microscale movement through the porous structure was observed in detail by using X-ray microimaging technique. The phenomenon was also analyzed using a theoretical model. This study provides insights into the hydraulic role of perforation plates in embolism repair. Furthermore, the present xylem-inspired channel platform can be used in various works on plant hydrodynamics.

## Materials and Methods

### Xylem-Inspired Channel With Embedded Mesh Structure

The xylem-inspired channel model comprises a straight channel embedded with a porous mesh structure (Figure 1B). This channel model is an open circular channel fabricated using a Tygon tube with an outer diameter of *d* = 0.7 mm as a channel mold (Mannino et al., 2015). The tube was tightly fixed with tension to form a straight line at the middle height of a Petri dish. Poly-dimethylsiloxane (PDMS) was partially cured using a 10:1 mixture of Sylgard-184 (Dow Corning) in the dish for 80 min at 50°C. The Tygon tube was then pulled out from PDMS. A piece of nylon mesh (Nylon mesh 100, APEC Industrial, Ltd.) was inserted between two excised cross sections of the circular PDMS channel. The fiber diameter and average opening width of the porous mesh were 100 and 150 μm, respectively. The contact angles of various kinds of Nylon were measured in the range from 74° to 94° (Fort, 1964). The wettability of Nylon reflects hydrophobic property of lignin that constructs various kinds of supporting tissues of xylem vessels (McCully et al., 2014). The excised cross-sectional channel surfaces were firmly adhered onto the mesh, and the channel model was completely cured for more than 2 h at 50°C. Based on the diameter of xylem vessels (Olson and Rosell, 2013; Rosell et al., 2017), the diameter of the xylem-inspired channel was scaled up by a factor of 10^0^ to 10^2^.

### Characteristic Flow Conditions

The water flow in the channel was supplied using a syringe pump (neMESYS 290N, Cetoni GmbH) with varying flow velocity *v* from 0.2 to 0.8 mm/min. Rhodamine-B solution (Acros Organics) was diluted in distilled water to a concentration of 50 mg/l and used as working fluid for observing the movement of the air–water meniscus. The ratio of inertia force to viscous force, prescribed by the Reynolds number *Re* = ρ*vd*/μ, was adjusted to the order of 10^−1^ in consideration of sap flow in xylem vessels (Jensen et al., 2016). Herein, ρ and μ denote the density and viscosity of the working fluid. Capillary number *Ca* = μ*v*/γ, the ratio of viscous force to surface tension, was additionally modulated to be comparable with that of real sap flow in the range of 10^−5^ (Jensen et al., 2016). Here, γ is the surface tension of water. The syringe contained 0.05 ml of air to regulate the internal pressure gradually.

### Quantitative Analysis of the Water-Filling Process

During water-filling in the channel model, the movement of each air–water meniscus was sequentially captured by a high-speed camera (pco 1200 hs, PCO) at a frame rate of 10 fps. The position of the meniscus was determined by reaching the maximum location in the pixel intensity gradient. The temporal variation of the moving velocity of the meniscus was analyzed using ImageJ software (Rueden et al., 2017). The pressure in front of the mesh structure was measured with a pressure transducer (PX409-015GUSB, Omega engineering). The penetration process of the air–water meniscus through the mesh was visualized by synchrotron X-ray microscopy at the 6C Biomedical Imaging Beamline at Pohang Accelerator Laboratory (PAL, Pohang, Korea). The X-ray beam energy was modulated to 24 keV, and the sample-to-detector distance was fixed at 50 mm. X-ray images were consecutively captured at a frame rate of 10 fps by using a Zyla camera (Andor Zyla, Ireland) with a 10 × objective lens. The field of view of the images was 1.7 × 1.4 mm in size, and the corresponding spatial resolution was 0.65 μm per pixel. The xylem-inspired channel was mounted on a translation stage for vertical and horizontal positioning. The flow rate was remotely controlled to 4.62 μl/min by using a syringe pump (PHD 2000 and PHD Ultra, Harvard Apparatus). The contact line speed and contact angle of the air–water meniscus in contact with the mesh structure were estimated from the captured consecutive X-ray images.

## Results

### Three-Step Water-Refilling Process at the Porous Mesh Structure

**Figure 2:**
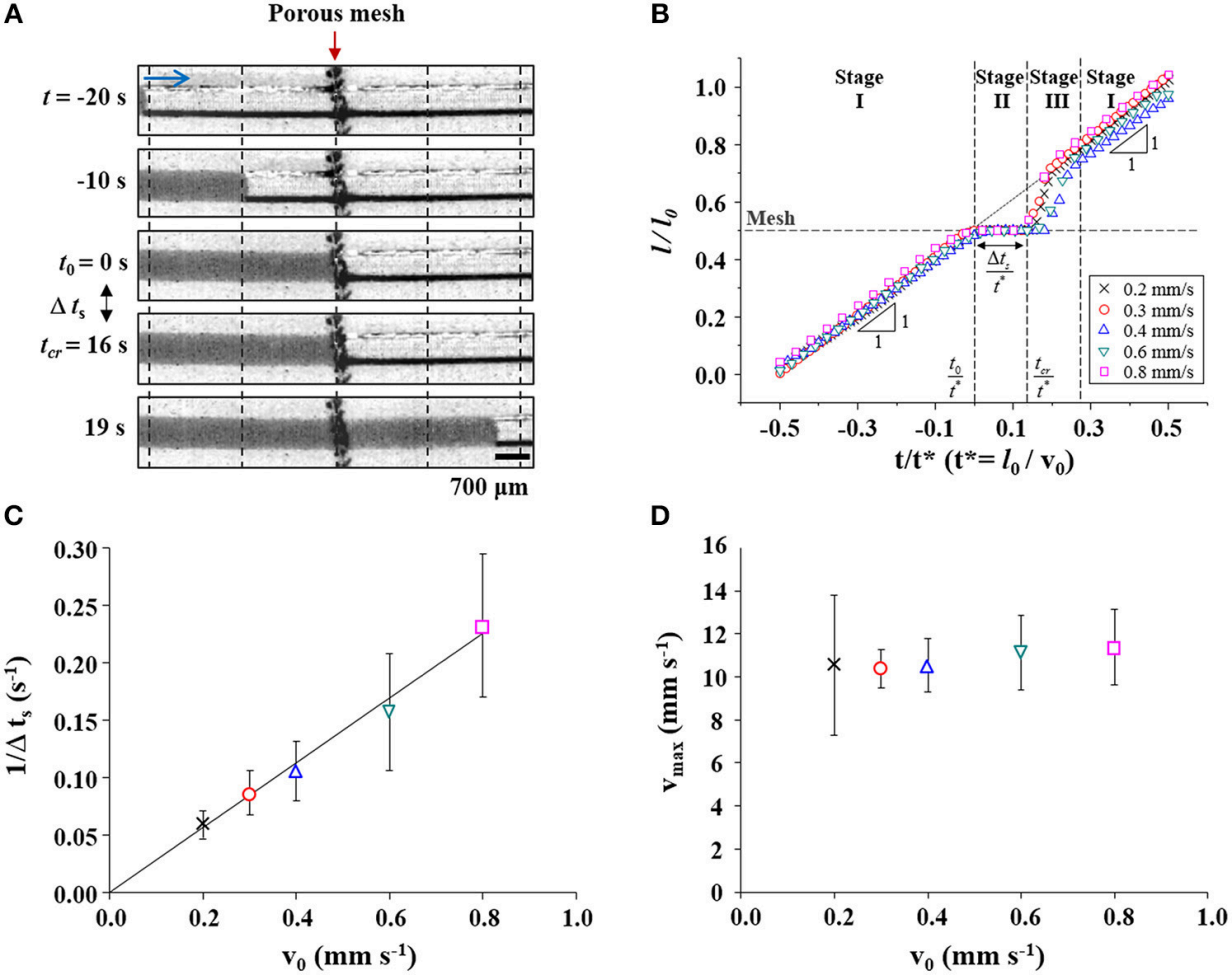
Flow characteristics of water-filling in the xylem-inspired channel. **(A)** Sequential images of the “stop (*t*_0_ ≤ *t* ≤ *t*_*cr*_) and acceleration (*t* > *t*_*cr*_)” pattern of the water meniscus when the water passes through the porous mesh at a flow velocity of *v*_0_ = 0.2 mm/s. Scale bar = 700 μm. **(B)** Normalized positional variations of the meniscus based on flow velocity. The “stop-and-acceleration” pattern of water-filling was classified into three stages. The mesh structure located at *l/l*_0_ = 0.5 is marked with a horizontal dashed line. Cross, circle, triangle, inverted triangle, and square symbols indicate the positions of the meniscus at different flow velocities *v*_0_ of 0.2, 0.3, 0.4, 0.6, and 0.8 mm/s, respectively. **(C)** The duration of water stoppage Δ*t*_*s*_ is inversely proportional to *v*_0_. The solid line is a linear fitting line (slope = 0.282 mm^−1^, R-squared value = 0.997). **(D)** Maximum water-refilling velocity according to initial velocity.

The water-filling process in the bioinspired channel embedding a porous membrane structure was investigated by matching its characteristic numbers, *Re* and *Ca*, to real sap flow in xylem vessels of vascular plants (Figure 2A). We monitored the movement of the water plug which appeared as a dark region in the time-sequential images. It moved from the left side to the right side of the channel. The In the initial 20 s (−20 < *t* < 0 s), the water plug moved at a constant speed, as shown by the images obtained every 10 s. The spacing between vertical dotted lines was constant. At *t* = 0 s, the meniscus reached the porous mesh embedded at the middle of the channel. We defined the time at which the meniscus touched the mesh as *t*_0_. The water meniscus stopped at the mesh for 16 s and then restarted toward the right side. The flow was accelerated immediately after passing through the membrane from *t* = 16 s to *t* = 19 s. The time of meniscus acceleration was defined as *t*_*cr*_, and the time duration of meniscus stoppage was expressed as Δ*t*_*s*_. This flow passing through the mesh structure exhibited a stop-and-acceleration pattern similar to the water-refilling phenomenon at perforation plates in the embolized xylem vessels (Jeje and Zimmermann, 1979; Lee and Kim, 2008; Kim and Lee, 2010; Lee et al., 2013; Hwang et al., 2016).

Positional variations of the meniscus during the water-filling process in the xylem-inspired channel were analyzed with varying initial flow velocity *v*_0_ (Figure 2B). The positional information of the meniscus and the time are expressed as dimensionless parameters *L* = *l*/*l*_0_ and *T* = *t*/t^*^, where *l* is the length of the water column inside the channel, *l*_*o*_ is the channel length, and t^*^ is the characteristic time *l*_0_/*v*_0_, respectively. The normalized positional variation of water, when *v*_0_ is 0.2 mm/s, clearly exhibit the stop-and-acceleration flow pattern at the porous mesh. The stop-and-acceleration flow pattern can be classified into three stages on the basis of the characteristic times *t*_0_ and *t*_*cr*_. In the first stage (stage I; during *t* < *t*_0_), the meniscus approached the mesh located at 0.5*l*_0_ with a constant speed *v*_0_. This result indicates water flow in a simple straight channel without any porous structure. In the next stage (stage II; *t*_0_ ≤ *t* ≤ *t*_*cr*_), the meniscus stopped at the mesh during Δ*t*_*s*_, after the meniscus came in contact with the mesh (*t* = *t*_0_). As illustrated in Figure 2C, the stoppage time Δ*t*_*s*_ was inversely proportional to *v*_0_. The solid line is the linear fitting line with a slope of 0.282, and the corresponding R-squared value is 0.997. In stage III (*t* > *t*_*cr*_), the meniscus completely passed the mesh and accelerated toward the right side. The maximum instantaneous velocity *v*_max_ was nearly invariant regardless of *v*_0_ (Figure 2D). The accelerated water meniscus recovered the original velocity *v*_0_ at the end of stage III. The flow pattern returned to stage I, where water-filling was negligibly affected by the presence of a porous mesh.

**Figure 3:**
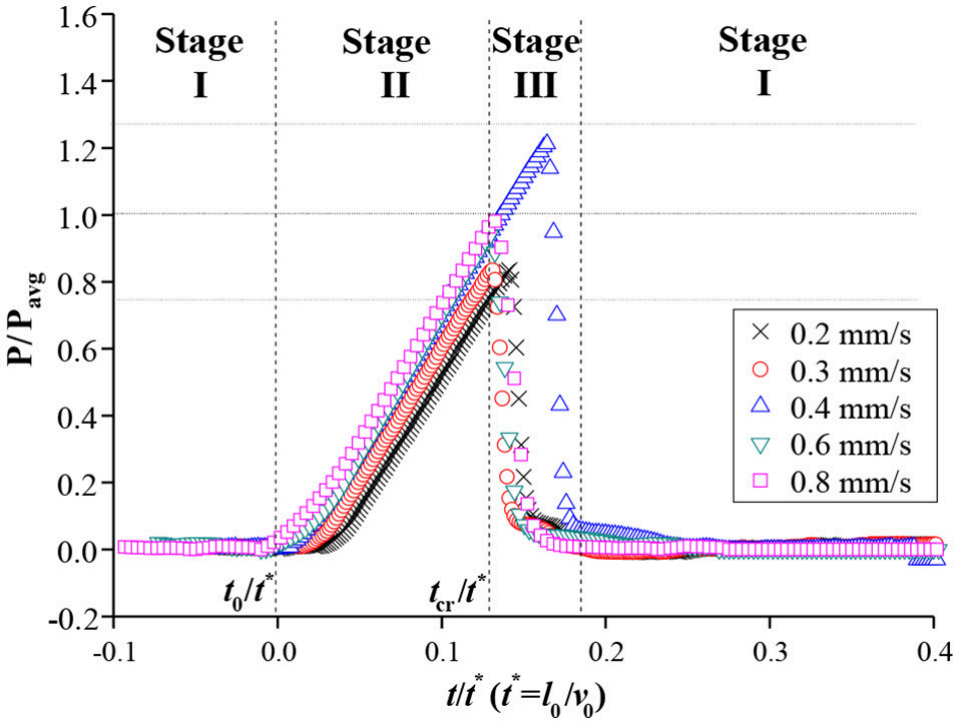
Normalized pressure variations in front of the mesh. Before the meniscus reached the mesh, pressure was maintained as the atmospheric pressure (stage I). After the meniscus met the mesh, pressure was maximized up to a certain range within ±25% of *P*_*avg*_ in stage II and then rapidly released to the atmospheric pressure at stage III.

The temporal variations in pressure *P* at the front side of the mesh were also measured during the water-filling process in the xylem-inspired channel. The three different stages were also clearly discerned in the graph of normalized *P* vs. *t* (Figure 3). The normalized pressure values were obtained by dividing *P* by the *P*_*avg*_ which is defined as the average of maximum critical pressures of each flow rate cases, and *t* by t^*^, respectively. As the meniscus approached the mesh at *t* < *t*_0_, the pressure was remained close to the atmospheric pressure (stage I). When the meniscus reached the mesh at *t* = *t*_0_, the pressure began to increase linearly, and the increasing rate was proportional to *v*_0_ in stage II (*t*_0_ ≤ *t* ≤ *t*_*cr*_). After reaching maximum pressures ranged from 0.75 to 1.25 *P*_*avg*_, the maximum pressure was released to the value near the atmospheric pressure. At this point, the meniscus completely passed through the mesh (stage III; *t* > *t*_*cr*_). The pressure was returned close to the atmospheric pressure, which corresponded to stage I.

### Dynamic Behavior of the Water Meniscus Passing Through the Porous Mesh

**Figure 4:**
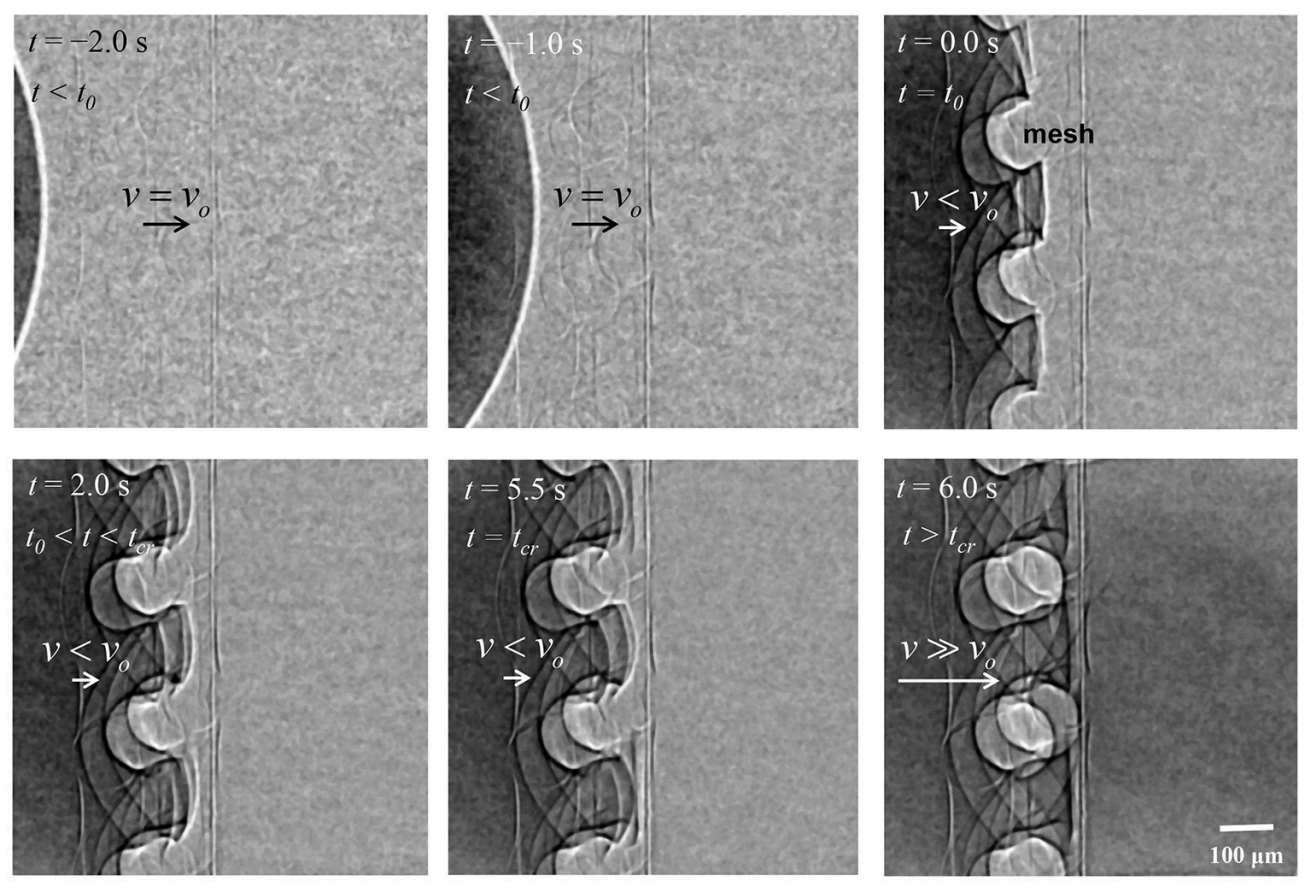
Typical X-ray images showing the water-filling flow in the xylem-inspired channel. Side view shows the movement of the water meniscus near the mesh structure. The water meniscus moved at a constant velocity *v*_0_ upon contact with the mesh structure at *t* = *t*_0._ The meniscus then split into smaller menisci between mesh pores. The menisci penetrated pores with a velocity lower than *v*_0_ and with increasing curvature until *t* = *t*_*cr*._ After the complete penetration of the water meniscus through the porous mesh, the menisci merged into a large meniscus, which then moved rapidly to the right side (*t* > *t*_*cr*)_. Scale bar = 100 μm.

The dynamic behavior of the water meniscus through the mesh structure was visualized by X-ray imaging technique (Figure 4, Supplementary Video S1). Phase-contrast X-ray imaging technique enables the clear visualization of the interfaces between different phases inside the opaque porous structures. The method also allowed the analysis of the variations in the contact line and meniscus curvature (Figure 4, Supplementary Video S1) and contact angles of the menisci (Supplementary Information S1). The captured consecutive X-ray images show the dynamic motion of the water plug from the left side to the right side. Each arrow indicates the vector of flow velocity *v*. Before the water plug reached the porous mesh (stage I; *t* < *t*_0_), the water meniscus moved with a constant velocity *v*_0_ toward the mesh located at the middle of each image. After the meniscus reached contact with the mesh at *t* = *t*_0_(= 0 s), the meniscus split into several smaller menisci to penetrate the mesh pores. When the water plug was anchored to the mesh (stage II; *t*_0_ ≤ *t* ≤ *t*_*cr*_), the menisci penetrated the mesh fibers with a lower velocity *v* compared with the initial velocity *v*_0_. Meanwhile, the curvatures of the convex-shaped menisci were increased until the end of stage II (*t* = *t*_*cr*_ = 4.5 s). Immediately before the start of acceleration (*t* = *t*_*cr*_), the contact angle between the water meniscus and the mesh fiber surface was measured to be 109.8° on average (Supplementary Information S1). Beyond the critical time *t*_*cr*_, the menisci burst through the porous mesh. The menisci then instantaneously merged into a meniscus, which accelerated beyond *v*_0_ toward the right side of the field of view for a short interval of 0.5 s (stage III; *t* > *t*_*cr*_).

## Discussion

On the basis of the experimental results, the stop (stage II; *t*_0_ ≤ *t* ≤ *t*_*cr*_) and acceleration (stage III; *t* > *t*_*cr*_) phenomenon of the meniscus at the mesh was analyzed. In stage I (*t* < *t*_0_), the meniscus advanced with a constant velocity of *v*_0_. Thus, this part was not theoretically analyzed because it reflects the water-filling flow in a simple channel without any structural barrier.

### Penetration of the Water Meniscus Through the Mesh Fibers at Stage II

**Figure 5:**
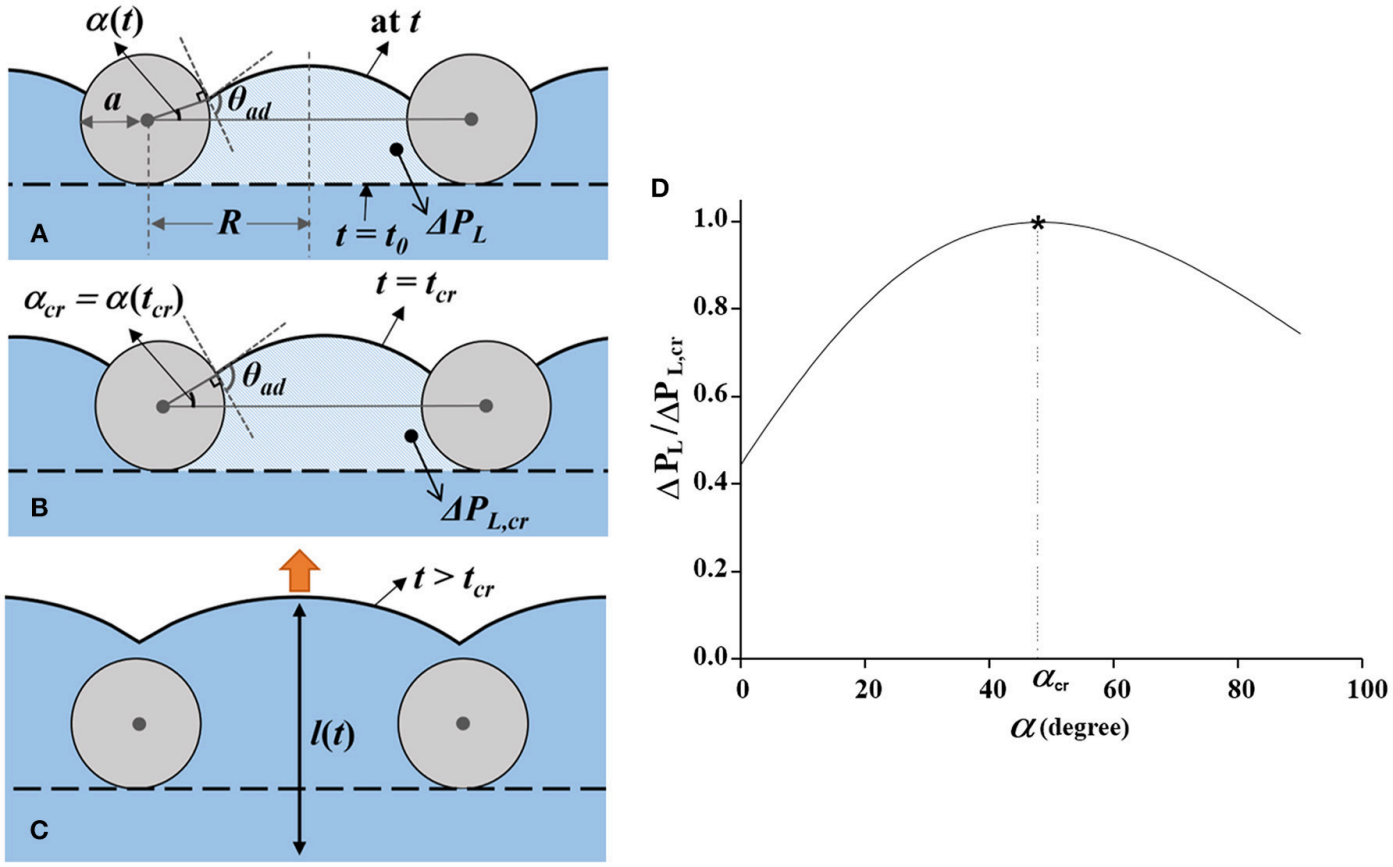
Dynamic movement of the water meniscus passing through a mesh pore. **(A)** Movement of the water meniscus during *t*_0_ ≤ *t* < *t*_*cr*._ From *t* = *t*_0_, when the meniscus is in contact with the mesh (horizontal dashed line), it gradually moves upward. The contact line of the meniscus moves along the surface of the mesh fibers with increasing α, the angle between the gray horizontal line and the contact position of the meniscus. The advancing contact angle of the meniscus is nearly maintained as θ_*ad*_. The distance between the centers of two adjacent mesh fibers and the radius of the mesh fiber are denoted as 2*R* and *a*, respectively. **(B)** Water meniscus at *t* = *t*_*cr*_. At this moment, α reaches α _cr_ and Δ*P*_*L*_ is maximized to Δ*P*_*L*__, cr_. **(C)** Acceleration of the water meniscus in stage III (*t* > *t*_*cr*_). When the characteristic angle α exceeds α_*cr*_, the water flow accelerates by Δ*P*_*L*__, cr._ **(D)** Variation of normalized Laplace pressure as a function of α (*t*). The pressure increases up to Δ*P*_*L*, cr_ at *t* = *t*_*cr*_, immediately before the flow acceleration.

The penetration process of the water meniscus through the mesh pores in stage II is simplified in Figure 5A. To represent the pressure increase caused by meniscus advancement, we defined an angle α between the horizontal line and the tangent line of the meniscus curve at the mesh fiber. The shapes of the meniscus at *t* = *t*_0_ and *t* = *t*_*cr*_ are indicated by a black dotted line and a solid line, respectively. The angle α increases, as the contact line of the meniscus curve moves upward along the mesh fiber surface in stage II and the pressure difference across the interface increases according to the formula expressed as follows:

(1) $$\begin{array}{r} \Delta P_{L}=\frac{2\gamma \sin\left(\theta_{ad}+\alpha -\pi /2\right)}{\xi R-\zeta a\cos\alpha }=-\frac{2\gamma \cos\left(\alpha +\theta_{ad}\right)}{R_{eff}}, \end{array}$$

where θ_*ad*_ is the contact angle of the water meniscus advancing along the mesh fiber surface. The distance between the centers of two mesh fibers, and the radius of the mesh fiber are denoted as 2*R* and *a*, respectively. The average moving velocity of the contact line was measured to be 15 μm/s from the captured consecutive X-ray images. Since *Ca* is in the order of 10^−8^, the effect of contact angle variation was negligible (Berthier, 2008). Thus, the advancing contact angle θ_*ad*_ was assumed to be constant as the measured value of 109.8 ± 7.3° (Supplementary Information S1). In the above-mentioned equation, the effective radius of the mesh pore *R*_*eff*_ = ξ*R*−ζ*a*cosα was introduced to compensate for the shape effects of the actual mesh. The shape factor ξ reflects the rectangular shape and pore size irregularity which related to the pore size *R* and ζ modifies the woven structure of the mesh and squashing of mesh fiber that affect *a*. According to Equation (1), the pressure difference Δ*P*_*L*_ varies with α as shown in Figure 5D. It increases up to the maximum value of Δ*P*_*L, cr*_ as α increases to the critical angle α_*cr*_ at *t* = *t*_*cr*_ (Figure 5B). When s is maximized as Δ*P*_*L, cr*_, the derivative of Equation (1) with respect to α becomes zero at *t* = *t*_*cr*_ as follows:

(2) $$\begin{array}{r} {\frac{d\Delta P_{L}}{d\alpha }|}_{\alpha =\alpha_{cr}}=\frac{2\gamma }{{R_{eff}}^{2}}\left[\xi R\sin\left(\theta_{ad}+\alpha_{cr}\right)-\zeta a\sin\theta_{ad}\right]=0, \end{array}$$

(3) $$\begin{array}{r} \sin\left(\theta_{ad}+\alpha_{cr}\right)=\frac{\zeta a}{\xi R}\sin\left(\theta_{ad}\right). \end{array}$$

According to Equations (1) and (3), theoretical value of Δ*P*_*L, cr*_ and α_*cr*_ were thoroughly matched with the experimental result, 346.7 Pa and 35.7°, respectively, when ξ is about 5.4 and ζ is 8.1. The experimental value of α_*cr*_ was measured on the basis of the captured X-ray images. Detailed derivation of Equations (1–3) is provided in the Supplementary Information S2.

In the proposed model, the critical Laplace pressure Δ*P*_*L, cr*_, at which the water begins to pass through the porous mesh does not depend on *v*_0_. This result supports that the maximum pressure remained within a certain range, regardless of the initial flow velocity (Figure 3). Because the Δ*P*_*L, cr*_ is not a function of *v*_0_, the case of water-refilling at a faster *v*_0_ reaches the critical state Δ*P*_*L, cr*_, and α_*cr*_ faster. This patterns implies that the pressure increasing rate is proportional to *v*_0_, and Δ*t*_*s*_ is inversely proportional to *v*_0_ (Figure 2C). Moreover, under the proposed model, the critical Laplace pressure Δ*P*_*L, cr*_ increases with decreasing pore size and wettability of the porous mesh.

### Flow Acceleration at Stage III

In stage III, the menisci that formed at each pore merged into a meniscus, which was then accelerated forward (Figure 5C). The flow acceleration at this stage can be mathematically described by balancing the inertial, capillary, viscous pressures, and the pressure drop caused by the hydraulic resistance of the mesh structure (*R*_*m*_). At the instant of flow acceleration, the position of the water meniscus *l*(*t*) is expressed as

(4) $$\begin{array}{r} \rho \frac{d}{dt}\left(l\frac{dl}{dt}\right)=\Delta P_{L,cr}-\frac{8\mu }{r^{2}}l\frac{dl}{dt}-\pi r^{2}R_{m}\frac{dl}{dt}. \end{array}$$

where *r* is the radius of the circular channel. When a water meniscus passes through the porous mesh, the pressure in front of the mesh increases up to the maximum Laplace pressure Δ*P*_*L, cr*_. As the pressure increases to the maximum value of Δ*P*_*L, cr*_, flow acceleration is driven by Δ*P*_*L, cr*_ and the pressure is instantaneously released to the atmospheric pressure. By contrast, the viscous force and pressure drop caused by hydraulic resistance of the mesh suppress the flow acceleration as resisting force. Through the dimensionless parameters *L* = *l*/*l*_0_, *T* = *t*/t^*^, and β = *l*_*o*_/*r*, Equation (4) is transformed into

(5) $$\begin{array}{r} \frac{d}{dT}\left(L\frac{dL}{dT}\right)=\frac{\Delta P_{L,cr}}{\rho {v_{0}}^{2}}-\frac{8\beta }{Re}L\frac{dL}{dT}-\frac{\Delta P_{m}}{\rho {v_{0}}^{2}}\frac{dL}{dT}, \end{array}$$

where $\Delta P_{m}=\pi r^{2}v_{o}R_{m}$ denotes the pressure drop caused by *R*_*m*_. The values of *Re* for the given initial flow velocities are in the range of 0.07–0.28. The average Δ*P*_*L, cr*_ was experimentally obtained to be 346.7 Pa, β and $\pi r^{2}R_{m}$ are 57.1 and 4335.5 Pa s/m, respectively.

At the initial phase of stage III (*t* = *t*_*cr*_), the water meniscus burst from the mesh pores. Flow was accelerated by the accumulated maximum pressure difference Δ*P*_*L, cr*_ formed at the meniscus. Then, the flow decelerated to the initial velocity *v*_0_ because of viscous dissipation and the hydraulic resistance *R*_*m*_. Since the magnitudes of the terms representing Δ*P*_*L, cr*_, the viscous force and the hydraulic resistance of the mesh exceed 10^3^, the other term is negligible. In addition, the derivative *dL*/*dT* is assumed to be *V*_max_ = *v*_max_/*v*_0_ at the initial stage of the flow acceleration. In this regard, Equation (5) can be simplified as

(6) $$\begin{array}{r} 0=\frac{\Delta P_{L,cr}}{\rho {v_{0}}^{2}}-\frac{8\beta }{Re}L_{0}V_{\max}-\frac{\Delta P_{m}}{\rho {v_{0}}^{2}}V_{\max}, \end{array}$$

where *L*_0_ is the dimensionless length of the water plug during the acceleration. From Equation (6), the relationship of $V_{\max}\sim {v_{0}}^{-1}$ is obtained. This relation supports that *v*_max_ is constant, regardless of *v*_*o*_ (Figure 2D). More detailed derivation of Equations (4–6) is provided in the Supplementary Information S3.

Water-refilling flows that pass through perforation plates in embolized xylem vessels have been reported to exhibit the stop-and-acceleration pattern. However, the hydraulic functions of perforation plates have not been thoroughly examined with the aid of theoretical support. The present experimental and theoretical investigations are thus helpful for understanding the hydraulic roles in terms of water-refilling and pressure regulation inside embolized xylem vessels. The refilling water plug begins to advance into pores of the mesh structure and appeared to cease moving at the mesh. However, in our real-time microscopic view, the menisci formed between the mesh fibers advanced continuously and immediately accelerated when the contact angle reached the critical value. The geometrical features of the mesh structure determined the critical pressure operating as a resisting force against water-filling flow. The pressure in the compartment filled with water was gradually increased up to the threshold value. The critical pressure difference also served as a driving force to instantaneously accelerate the water-filling flow.

These results imply that perforation plates in xylem vessels may function as hydraulic valves that raise the hydrostatic pressure inside the vessel compartment during water-refilling in embolized xylem vessels (Lee and Kim, 2008; Hwang et al., 2014). In this process, the refilling vessel could be pressurized when the meniscus passes the perforation plates. The pressure increase at the porous structures may help dissolve gas bubbles into the water inside the embolized xylem vessels (Tyree and Yang, 1992; Schenk et al., 2016). Scholars conjectured that the porous structures in xylem vessels briefly hinder axial water-refilling and then simultaneously increase the local pressure to enable radial water-refilling flow (Hwang et al., 2016).

Through the xylem-inspired channel composed of a channel embedded with a porous structure, the characteristics of water-refilling flow around a porous mesh were systematically examined. Our experimental and theoretical analyses were limited to a regularly shaped porous structure with large-scale pores and the pressure inside the xylem-inspired channel has a large difference with the pressure inside xylem vessels. However, this study can still contribute to the understanding of the hydraulic functions of perforation plates for embolism repair. The proposed xylem-inspired channel model can be also applied to validate many hypotheses on embolism repair and plant hydrodynamics.

## Author Contributions

SL, JP, and JR proposed and designed the study. JP conducted the experiments. JP and JR analyzed the experimental data. All authors discussed the results and wrote the manuscript.

### Conflict of Interest Statement

The authors declare that the research was conducted in the absence of any commercial or financial relationships that could be construed as a potential conflict of interest.

## Supplementary Material

Supplementary Information S1 Measurement of advancing contact angle θ _*ad*_ by using the X-ray images during the water meniscus passing through the mesh structure.

Supplementary Information S2 Detailed derivation of equations 1–3 for the meniscus penetration through the mesh.

Supplementary Information S3 Detailed derivation of equations 4–6 for the flow acceleration.

Supplementary Video S1 Dynamic behavior of the water meniscus passing through the porous mesh.
