# Supplementary material for: Hydrodynamic Study on the “Stop-and-Acceleration” Pattern of Refilling Flow at Perforation Plates by Using a Xylem-Inspired Channel
Source: Front Plant Sci. 2019 Jan 8;9:1931. doi: 10.3389/fpls.2018.01931 (PMC6331423; doi:10.3389/fpls.2018.01931)
Supplement: Supplementary Information S1 — Measurement of advancing contact angle θ ad by using the X-ray images during the water meniscus passing through the mesh structure. [file Data_Sheet_1.pdf]

Supplementary Information S1| Measurement of the advancing contact angle θ_{ad} by using the X-ray images during the water meniscus passing through the mesh structure. Yellow circle indicates cross-sectional view of the mesh fiber. Blue curve is the water meniscus. Several θ_{ad} values were measured and they were averaged as 109.8±7.3°. Scale bar = 30 μm.

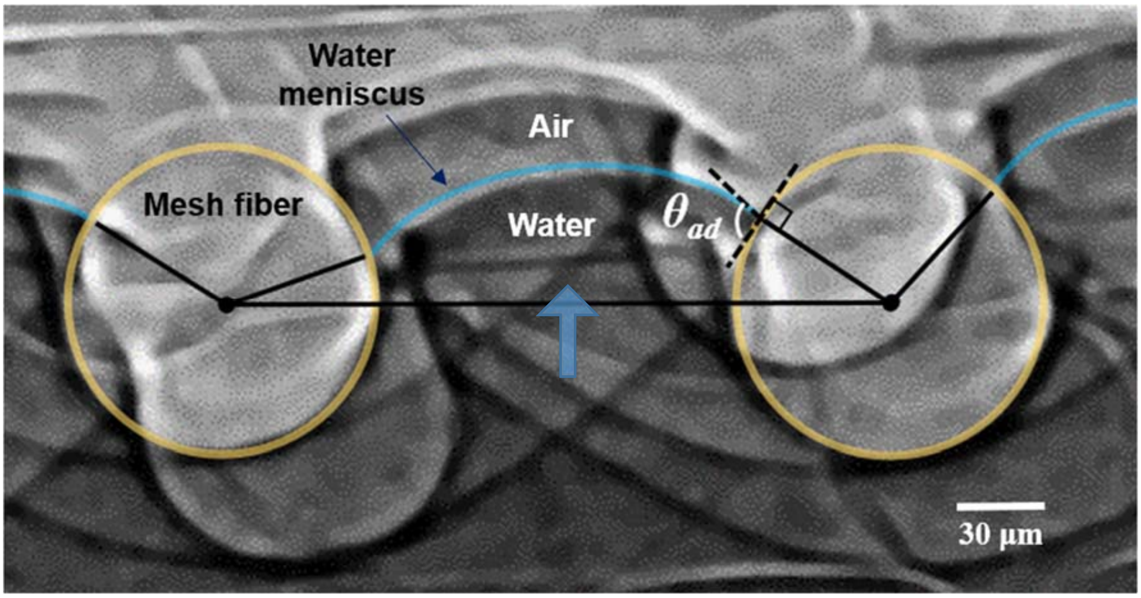

Supplementary Information S2| Detailed derivation of Eqs. 1−3 for the meniscus penetration through the mesh.

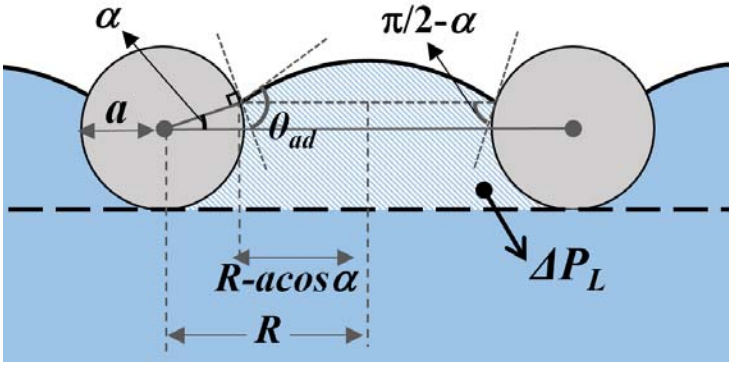

Laplace pressure ΔP_L exerted on the air-water interface passing the mesh pore is expressed as follows:

$$\Delta P_L = \frac{2\gamma \sin(\theta_{ad} - (2/\pi - \alpha))}{R - a\cos\alpha} = -\frac{2\gamma \cos(\alpha + \theta_{ad})}{R - a\cos\alpha} \tag{s1}$$

where θ_{ad} is the contact angle of the water meniscus advancing along the mesh fiber surface. The distance between the centers of two adjacent mesh fibers, and the radius of the mesh fiber are denoted as $2R$ and a, respectively. The angle between the horizontal line and the tangent line of the meniscus curve at the mesh fiber is denoted as α. By considering the irregular shape of the mesh pore and mesh fiber, shape factors ξ and ζ are multiplied to R and radius of mesh fiber a. With introducing the effective radius of the mesh pore $R_{eff} = \xi R - \zeta \cos\alpha$, Eq. (s1) becomes

$$\Delta P_L = -\frac{2\gamma \cos(\alpha + \theta_{ad})}{\xi R - \zeta a\cos\alpha} = -\frac{2\gamma \cos(\alpha + \theta_{ad})}{R_{eff}}. \tag{s2}$$

To find $\Delta P_{L,cr}$ and α_{cr}, where ΔP_L is maximized, Eq. (s2) is differentiated as

$$\begin{aligned}\frac{d\Delta P_L}{d\alpha} &= \frac{2\gamma}{R_{eff}^{\ 2}}\left[\sin(\alpha+\theta_{ad})(\xi \mathrm{R} - \zeta a\cos\alpha) + \cos(\alpha+\theta_{ad})\zeta a\sin\alpha\right] \\ &= \frac{2\gamma}{R_{eff}^{\ 2}}\left[\xi \mathrm{R}\sin(\alpha+\theta_{ad}) - \zeta a(\cos\alpha\sin(\alpha+\theta_{ad}) - \cos(\alpha+\theta_{ad})\sin\alpha)\right] = 0.\end{aligned} \tag{s3}$$

By applying trigonometric formula,

$$\begin{aligned}\frac{d\Delta P_L}{d\alpha} &= \frac{2\gamma}{R_{eff}^{\ 2}}\left[\xi \mathrm{R}\sin(\alpha+\theta_{ad}) - \zeta a\sin((\alpha+\theta_{ad}) - \alpha)\right] \\ &= \frac{2\gamma}{R_{eff}^{\ 2}}\left[\xi \mathrm{R}\sin(\alpha+\theta_{ad}) - \zeta a\sin\theta_{ad}\right] = 0.\end{aligned} \tag{s4}$$

Therefore, ΔP_L is maximized, when α_{cr} satisfies the following equation,

$$\sin(\theta_{ad} + \alpha_{cr}) = \frac{\zeta a}{\xi R}\sin(\theta_{ad}). \tag{s5}$$

Supplementary S3 | Detailed derivation of Eqs. 4–6 for the flow acceleration.

During the flow acceleration, the position of the water meniscus $l(t)$ is expressed as

$$\rho\frac{d}{dt}(l\frac{dl}{dt})=\Delta P_{L,\mathrm{cr}}-\frac{8\mu}{r^2}l\frac{dl}{dt}-\pi r^2 R_m\frac{dl}{dt} \tag{s6}$$

by balancing the inertial, capillary, viscous pressures, and the pressure drop caused by the hydraulic resistance of the mesh structure (R_m). Water density, viscosity, and the radius of the circular channel are denoted as ρ, μ, and r, respectively. By submitting dimensionless parameters $L=l/l_0$, $T=t/t^*$, $t^*=l_o/v_0$, and $\beta=l_o/r$, Eq (s6) becomes

$$\rho\frac{d}{d(Tt^*)}\mathrm{L}l_0\frac{d(Ll_0)}{d(Tt^*)}=\Delta P_{L,\mathrm{cr}}-\frac{8\mu}{r^2}Ll_0\frac{d(Ll_0)}{d(Tt^*)}-\pi r^2 R_m\frac{d(Ll_0)}{d(Tt^*)} \tag{s7−1}$$

$$\rho\frac{l_0^2}{t^{*2}}\frac{d}{dT}\left(\mathrm{L}\frac{dL}{dT}\right)=\Delta P_{L,\mathrm{cr}}-\frac{8\mu\beta v_0}{r}L\frac{dL}{dT}-\frac{\pi r^2 l_0 R_m}{t^*}\frac{dL}{dT} \tag{s7−2}$$

$$\frac{d}{dT}\left(\mathrm{L}\frac{dL}{dT}\right)=\frac{\Delta P_{L,\mathrm{cr}}}{\rho v_0^2}-\frac{8\mu\beta}{\rho v_0 r}L\frac{dL}{dT}-\frac{\pi r^2 R_m}{\rho v_0}\frac{dL}{dT} \tag{s7−3}$$

where v_0 is the initial flow velocity. The average $\Delta P_{L,cr}$ was experimentally obtained to be 346.7 Pa, β and $\pi r^2 R_m$ are 57.1 and 4335.5 Pa s/m, respectively. By introducing the Reynolds number $\mathrm{Re}=\rho v_o r/\mu<1$, and the pressure drop $\Delta P_m=\pi r^2 v_o R_m$ caused by R_m,

$$\frac{d}{dT}\left(\mathrm{L}\frac{dL}{dT}\right)=\frac{\Delta P_{L,\mathrm{cr}}}{\rho v_0^2}-\frac{8\beta}{\mathrm{Re}}L\frac{dL}{dT}-\frac{\Delta P_m}{\rho v_0^2}\frac{dL}{dT}. \tag{s8}$$

By considering the relative magnitudes of the coefficient of each terms, the inertial term on the left side becomes negligible. At the instant of flow acceleration, dL/dT is assumed to be $V_{\max}=v_{\max}/v_0$ and Eq. (s8) can be simplified as

$$0=\frac{\Delta P_{L,\mathrm{cr}}}{\rho v_0^2}-\frac{8\beta}{\mathrm{Re}}L_0 V_{\max}-\frac{\Delta P_m}{\rho v_0^2}V_{\max}, \tag{s9}$$

where L_0 is the dimensionless length of the water column during the flow acceleration.
